# Small Gap Dynamics in High Mountain Central European Spruce Forests—The Role of Standing Dead Trees in Gap Formation

**DOI:** 10.3390/plants13243502

**Published:** 2024-12-15

**Authors:** Denisa Sedmáková, Peter Jaloviar, Oľga Mišíková, Ladislav Šumichrast, Barbora Slováčková, Stanislav Kucbel, Jaroslav Vencurik, Michal Bosela, Róbert Sedmák

**Affiliations:** 1 Department of Silviculture, Faculty of Forestry, Technical University in Zvolen, T. G. Masaryka 24, 960 01 Zvolen, Slovak Republickucbel@tuzvo.sk (S.K.); vencurik@tuzvo.sk (J.V.); 2 Department of Wood Science, Faculty of Wood Sciences and Technology, Technical University in Zvolen, T. G. Masaryka 24, 960 01 Zvolen, Slovak Republic; 3 Department of Forest Resource Planning and Informatics, Faculty of Forestry, Technical University in Zvolen, T. G. Masaryka 24, 960 01 Zvolen, Slovak Republicsedmak@tuzvo.sk (R.S.)

**Keywords:** young gaps, deadwood dynamics, mortality, basic specific gravity, cell structure, timberline, soil protective management

## Abstract

Gap dynamics are driving many important processes in the development of temperate forest ecosystems. What remains largely unknown is how often the regeneration processes initialized by endogenous mortality of dominant and co-dominant canopy trees take place. We conducted a study in the high mountain forests of the Central Western Carpathians, naturally dominated by the Norway spruce. Based on the repeated forest inventories in two localities, we quantified the structure and amount of deadwood, as well as the associated mortality of standing dead canopy trees. We determined the basic specific gravity of wood and anatomical changes in the initial phase of wood decomposition. The approach for estimating the rate of gap formation and the number of canopy trees per unit area needed for intentional gap formation was formulated based on residence time analysis of three localities. The initial phase of gap formation (standing dead tree in the first decay class) had a narrow range of residence values, with a 90–95% probability that gap age was less than 10 or 13 years. Correspondingly, a relatively constant absolute number of 12 and 13 canopy spruce trees per hectare died standing in 10 years, with a mean diameter reaching 50–58 cm. Maximum diameters trees (70–80 cm) were represented by 1–4 stems per hectare. The values of the wood-specific gravity of standing trees were around 0.370–0.380 g.cm^−3^, and varied from 0.302 to 0.523 g.cm^−3^. Microscopically, our results point out that gap formation is a continuous long-lasting process, starting while canopy trees are living. We observed early signs of wood degradation and bacteria, possibly associated with bark beetles, that induce a strong effect when attacking living trees with vigorous defenses. New information about the initial phase of gap formation has provided a basis for the objective proposal of intervals and intensities of interventions, designed to promote a diversified structure and the long-term ecological stability of the mountain spruce stands in changing climate conditions.

## 1. Introduction

The death of one or more trees in the canopy leads to the creation of gaps, a common disturbance in many forest ecosystems [1,2,3]. Small canopy gaps are more commonly caused by external biotic agents or disturbance events rather than by endogenously controlled senescence, which only marginally affects the death of trees [4,5]. The trees die standing with the crown, and they may break or be uprooted [6,7]. In European temperate forests, the importance of small-scale tree mortality in old-growth forests was recognized early on [8,9], but studies primarily concentrated on describing the phases of forest development. Later Central European studies described complex gap disturbance regimes with gap sizes ranging from small (formed by the slow continuous death of single canopy trees) to larger (formed by episodic windstorm events) [10,11]. Among gap characteristics, gap age, defined as the “time since gap formation” [12], is likely to play an important role in gap dynamics, gap-filling processes or the identification of the phases in the canopy closing process [1,13,14]. There are several methods for estimating gap age, including repeated forest or remote sensing inventories [15,16], counting whorls of saplings [2], estimation from the degree of decomposition of the dead trees forming gaps [17], and growth analysis of trees surrounding or filling the gaps [18]; however, the accuracy of gap age estimation methods needs to be improved [19].

In high mountain spruce forests, trees mostly die standing, representing 50% or more of total coarse woody debris [17,20,21,22]. The spruce trees likely have longer crowns, which is reflected in their higher static stability. Most of the spruce trees therefore die standing, often because of multiple crown breaks caused by heavy snow, wind or frost, or are killed by bark beetle insects, which are more pronounced disturbing factors. The silvicultural prescription mimicking such mortality processes is single-tree selection [23,24]. Yet, the interval between single-tree disturbances, the forest area disturbed, and the sizes of dead canopy trees are not well known for Central European high mountain spruce forests, even though they are important parameters to maintain specific diameter distribution and maximum diameter under the single-tree selection system.

Time since death, along with other structural attributes of deadwood, are important and relevant indicators for close-to-nature silvicultural systems. Considering changes in volume, specific gravity in dead wood and specifically, in SDTs, can contribute to the more precise assessments and projections of carbon storage or biodiversity in mountain/boreal spruce forests. Despite the importance of standing dead trees (SDTs) in high mountain environments, very few studies have examined their attributes or dynamics [25,26,27,28]. The processes of decay have traditionally focused on lying wood [26,29,30]. Estimation of decomposition rates and residence times of trees in different decay classes is indicative of changes in deadwood amount and quality over time, and can be used for the estimation of gap age [25,31,32]. The rate of wood decay of dead trees has significant ecological and management implications. In this regard, a substantial portion of the carbon sequestrated by mountain forests is stored in SDTs [33]. The carbon stored in SDTs is not included in the rapid turnover because of the slow rate of wood decomposition (much lower compared to down/lying wood), thus, SDTs can be regarded as reservoirs of organic carbon, with a slow rate of its release back into the atmosphere [33,34,35]. 

Through decomposition, tree volume is lost and a reduction in specific gravity (also referred to as wood density [29,36]) occurs. For the estimation of the carbon stock in SDTs, species-specific gravity reduction factors are required, which then represent the index of the solid substance contained in a piece of dry wood [37,38]. Specific gravity values are obtained from cross-sections of a tree, or from increment cores. Generally, climate (mean temperature and moisture availability) is considered the primary control on the activity of decomposers (bacteria and fungi colonizing wood), but local scale factors (nitrogen availability, tree species identity, and stand conditions) can be more influential [39]. In managed coniferous forests, wood density depends on tree-ring width [40]. In even-aged Norway spruce stands, wood density increases along the stem radius, because of age-related long-term decreasing trends of tree-ring widths [41]. Uneven-aged trees, on the other hand, should display variable wood density profiles along the stem radius, as their growth is more influenced by individual growth releases and suppression [41]. Increased radial growth of the Norway spruce is usually associated with reduced tree-ring density, because of a reduction in latewood proportion [42,43]. Decreased radial growth can also be associated with lower rates of spruce wood decomposition [44], with decomposition rate depending on tree size as well [45].

To promote the understanding of the role of SDTs in small gap formation in high mountain Norway spruce forests, three representative sites were selected and measured in two mountain ranges in the Central Western Carpathians in Slovakia. The aims of our study are as follows:

- To enhance the information about the proportion (share) of large SDTs on total amount of deadwood (coarse woody debris) in spruce mountain stands;
- To provide a new piece of information about the mean residence time of canopy SDTs in the first decay class and their basic specific gravity compared with the specific gravity of living trees (LTs) growing at the same sites;
- To explore the relationships and variability between the mean residence time, basic specific gravity, and the age–size-related characteristics of SDTs in given conditions;
- To identify the wood anatomical changes of standing living and dead trees associated with the gap formation;
- To explore possible differences in relations of basic specific gravity and age–size-related characteristics between groups of SDTs and LTs, to reveal the possible causes of SDT mortality and the initiation of the gap formation.

All quantifications of the deadwood, time intervals, and intensity associated with the death of standing canopy trees in small gap formations in the natural conditions of high mountain Norway spruce forests can improve the effectivity of their tending, which is based on mimicking small-scale gap dynamics. In general, we aim to explore the properties of standing dead trees to improve gap age estimation and to promote the understanding of gap dynamics in the early stage of their formation in high mountain conditions. Moreover, we provide new information about a species-specific gravity in the decomposition process, and its relations to age–size tree variables that can be used for more accurate assessments or projections of biodiversity or carbon storage in the future.

## 2. Results

### 2.1. Deadwood Structure and Dynamics

The deadwood accounted for a substantial portion (two-fifths) of the total volume of living trees at both studied sites. The proportion of SDTs was higher on the Prasiva (PR) site (Table 1). 

**Figure 1:**
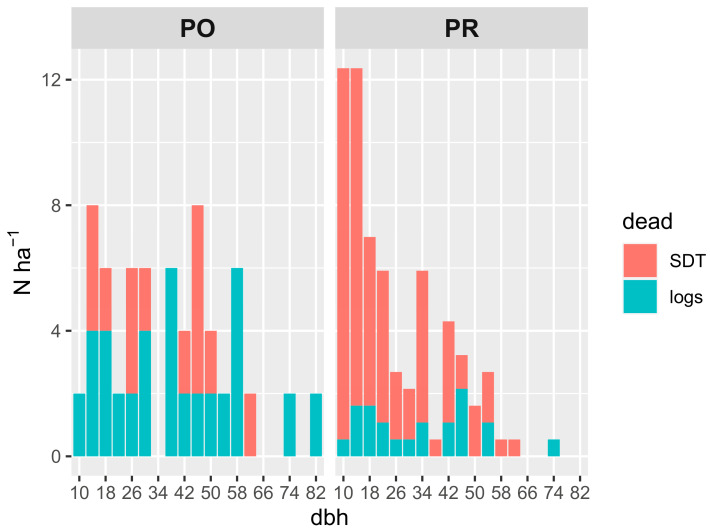
Mortality and structure of deadwood in high mountain spruce forests. Number of stems per hectare (Nha-1) according to diameter classes (DBH) on Polana (PO) site between 2013 and 2018 and on Prasiva (PR) site between 2007 and 2022. Number of stems was recalculated for a common 10-year period.

**Figure 2:**
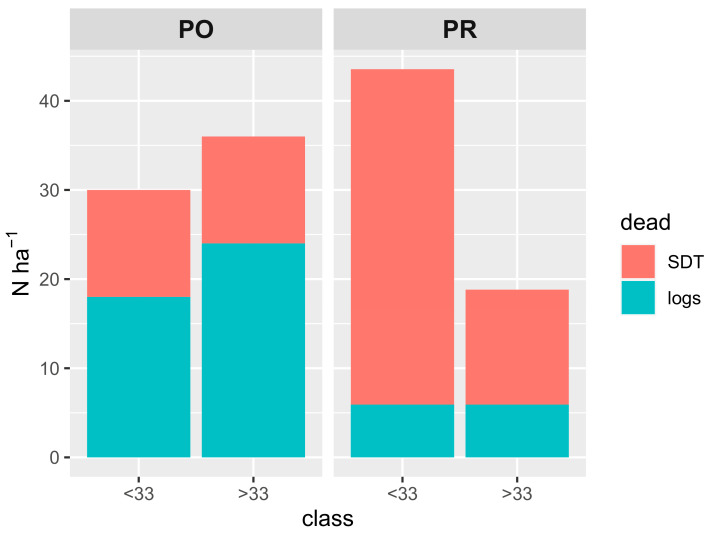
Mortality in high mountain spruce forests according to two tree layers, based on the DBH class. Number of stems per hectare (Nha^−1^) was recalculated for a common 10-year period.

In Poľana (PO) old-growth forest, 36% of the number of dead trees that died between the two inventory years 2013 and 2018 resulted in SDTs (6.7% of the volume and 7.2% of the basal area of LTs in 2013), while these trees accounted for 31% and 30% of the basal area and volume from total deadwood in the monitored period, respectively. After the recalculation, 24 trees per hectare died standing and 42 stems died and were overthrown, respectively, for a 10-year period. Mortality was evenly distributed throughout the diameter classes (Figure 1) and lying dead trees prevailed in both lower and upper tree layers defined by diameter classes under and over 33 cm (Figure 2). The border between the upper and lower tree layers was determined to be the minimum DBH of sampled SDTs, which was 33 cm for PO and PR.

In Prasiva (PR), 81% of the trees that died between 2007 and 2022 were SDTs. Their share on the basal area and volume from total deadwood was lower because of their considerably different proportion in the tree layers. The share of these trees on the basal area and volume was 6.7% of the volume and 9% of the basal area of LTs in 2007. Top diameter trees with a DBH above 70 cm were recorded as logs on both PO and PR sites and represented 1–4 stems per hectare. Based on the classification of deadwood into two layers according to the DBH class, the mortality in PR was significantly higher in the lower tree layers (Figure 1 and Figure 2), where we also observed a higher proportion of SDTs than in the upper canopy tree layers (Figure 2).

Regarding endogenous mortality in these high mountain spruce forests (occurring in upper diameter classes DBH > 33 cm), a similar absolute number of 12 and 13 canopy trees per hectare in 10 years died standing in PO and PR, respectively. Similarly, the total absolute number of dead trees per hectare in both localities was very similar—varying between 30–33 trees per hectare irrespective of the canopy layer. Nevertheless, the difference in the structure of deadwood (Figure 1) and in the proportions of standing and lying dead stems was obvious—in PO the ratio of standing to lying trees was approx. 1:2, whereas it was approx. 4:1 in PR. Thus, similar absolute numbers of dead trees in canopy position in both localities also results from different absolute mortality in tree layers; the PO site has a markedly higher mortality in the upper canopy layers (over 35 trees per hectare) than the PR site (with mortality under 20 trees per hectare). Such differences result from the fact that absolute mortality according to the layers is roughly equal at the PO site, but it is substantially uneven at the PR site. Here, 2/3 of dead trees came from lower layers, and only 1/3 from the upper ones. 

### 2.2. Living Trees (LTs) Chronologies

**Figure 3:**
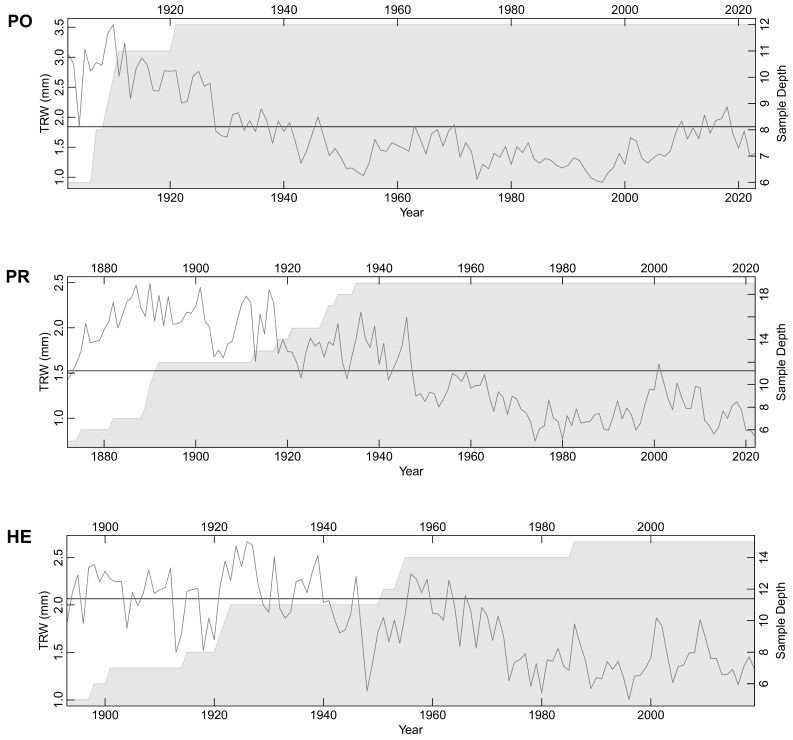
Mean tree-ring width (TRW) chronologies established from dominant living spruce trees for three localities (PO—Polana, PR—Prasiva, HE—Heľpa) to cross-date standing dead trees. Horizontal line corresponds with mean tree-ring width value. On the right y-axis, number of tree-ring series included to build master chronology is marked with grey color.

Three spruce master chronologies for PO, PR, and HE were obtained, extending up to 136 years (Figure 3). The PO chronology ranged from 1855 to 2023, the PR chronology from 1797 to 2022, and the HE chronology from 1864 to 2019. Tree-ring structure was easy to read; the average TRW values for PO, PR, and HE were 1.834 mm, 1.552 mm, and 2.073 mm, respectively. Tree-ring series were neither too sensitive nor complacent; the Gini coefficient for PO, PR, and HE was 0.243, 0.240, and 0.253, respectively. This enabled the successful construction of master chronologies for each locality. Each series included in the master chronology had a GLK (Gleichlaeufigkeit) value near 70% or higher (percentage of equivalent slope intervals).

### 2.3. Gapmakers—Standing Dead Trees (SDTs)

**Figure 4:**
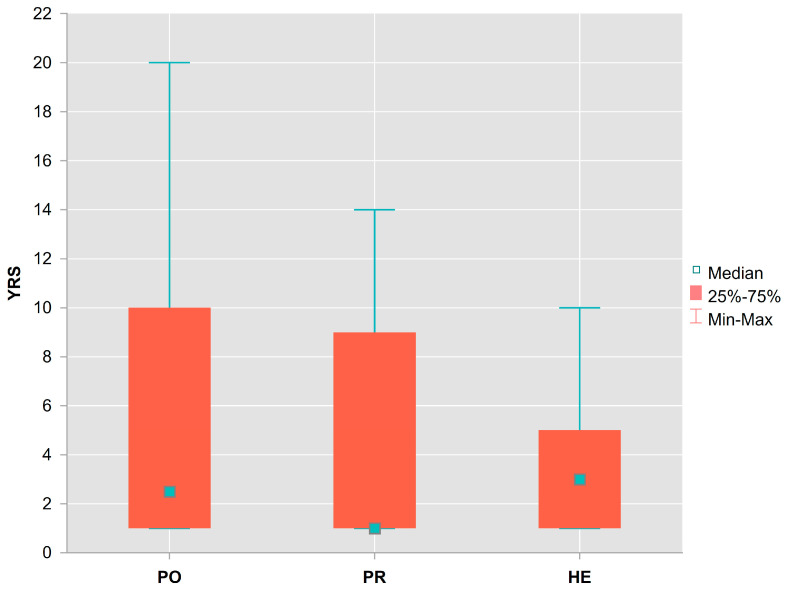
Number of years since death (YRS) for standing dead trees of the Norway spruce in the first decay class according to sampled localities Polana (PO), Prasiva (PR), and Helpa (HE).

All of the 38 sampled SDTs in the first decay class were cross-dated with site master chronologies. The one year since death refers to the calendar year in which the dead tree was sampled, and in which the last partial or complete ring was formed. The maximum number of 20 years since death was recorded on the PO site (Figure 4). 

Based on the Kruskal–Wallis test (H = 0.103 *p* < 0.950), the median value of years since death did not differ significantly between localities. Merged, the median time since death was 2.5 years.

**Figure 5:**
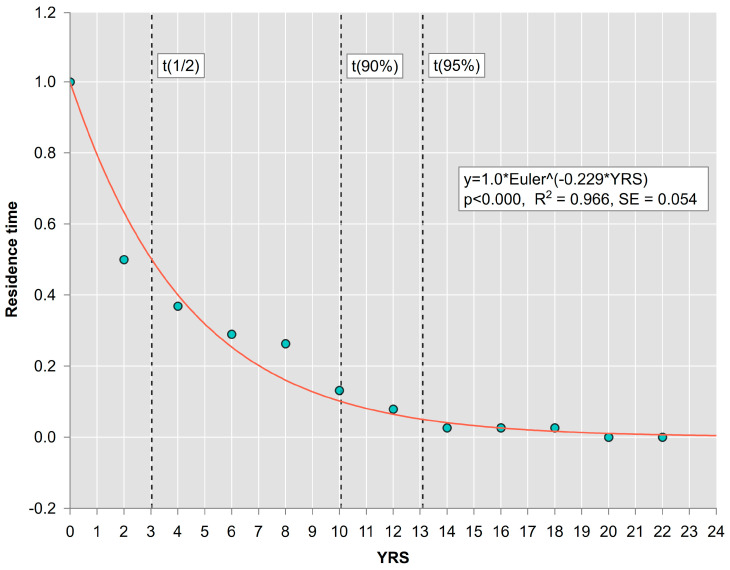
Predicted remaining fraction of the initial number of spruce trees, according to the number of years since tree death (YRS).

The relative frequencies of residence time of SDTs in the first decay class (calculated as the complement of empirical relative cumulative frequencies of times since death, Figure 5) were modelled by nonlinear exponential Equation (2). The model estimated residence constant k = −0.2287 (*p* < 0.000), which varied in 95% confidence limits from −0.266 to −0.192. The model performed well based on observed versus predicted values, and residuals fluctuated randomly around zero. The corresponding half residence time, calculated as t(1/2) = ln(0.5)/k, was 3.03 years. This means that SDTs of the spruce species remain in the first decay class for an average of 3 years, with a range of values between 2.6 and 3.6 years, with 95% of confidence. Approximately 90% of trees remain in the first decay class for about 10 years (with a range between 8.7 and 12.0 years). After 13 years, almost all trees drop out of the first decay class.

The residence time of dominant and co-dominant SDTs in the first decay class seemed to be related to stem age and size. Larger and older trees tended to reside (to persist) longer in the initial stage of gap formation, although the relationships were not statistically significant (*p* < 0.270 for AgeTotal, *p* < 0.228 for DBH), and accounted for less than 10% of the variance in residence time values (r = 0.27 for AgeTotal and r = 0.29 for DBH).

### 2.4. Basic Specific Gravity and Wood Anatomy of LTs and SDTs

For the sample of trees from the PO locality, we have determined the mean basic specific gravity ρ_B_ of SDTs and their counterpart LTs. We examined the differences in basic growth characteristics between these two groups of trees, as well the variation in basic specific gravity values along the radius, and its relationship with selected growth variables. There were no differences in the basic specific gravity of stems between dead and surrounding vital individuals of comparable age, dimensions, and social status (Table 2 and Table 3). The mean values of the basic specific gravity of standing trees were found to range from 0.302 g.cm^−3^ to 0.523 g.cm^−3^ under the given natural conditions. The basic specific gravity of spruce wood is 0.375 ± 0.052 g.cm^−3^ on average. 

**Figure 6:**
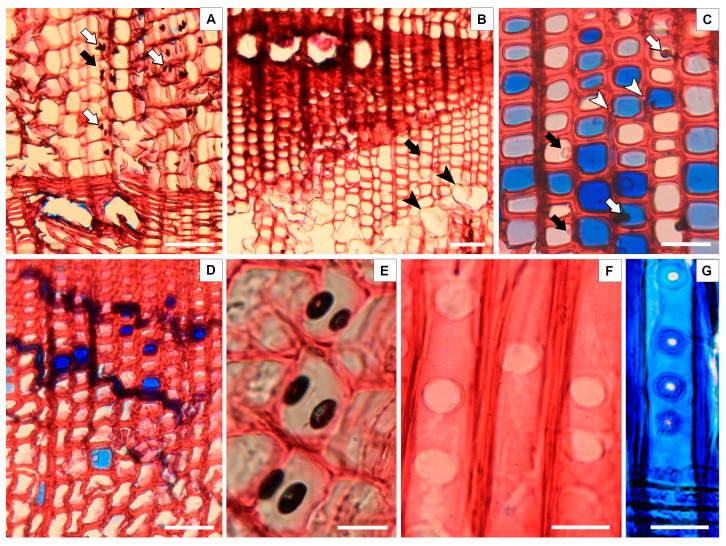
Figures (**A**–**C**) are healthy spruce trees H01 and H04, (**D**–**F**) are standing dead trees S01 and S04. Figure (**G**) shows a healthy bordered pit. (**A**–**E**) show a cross microsection of the spruce trees, F is a radial microsection. The scale of each figure is denoted with a white stripe in the lower right corner of the respective figures and is as follows: 100 μm in (**A**,**B**), 50 μm in (**C**,**D**), and 25 μm in (**E**–**G**). Black and white arrows mark traits described in the text below.

Figure 6 presents a microscopical analysis of the spruce trees. Macroscopically, the healthy wood seemingly did not have any signs of deterioration, but under the microscope, pathogen activity was obvious. Hyphae were not found in any of the samples, it is therefore suspected that it was a bacterial infection. We will refer to the pathogen as bacteria in the following text.

In Figure 6A, the beginning of the degradation process began in the vicinity of the many resin canals. The bacterial infection progressed through the ray parenchyma cell walls, where nutrients are stored. It entered the lumina of the earlywood tracheids through bordered pits. This process consumes the nutrients inside the cells and in the end reduces the flow of nutrients during the physiological processes in the tree. Figure 6B shows numerous resin canals in both earlywood and latewood. The resin canals in earlywood are considered traumatic resin canals, and are the result of the worsened living conditions of the tree. These traumatic resin canals are depicted in Figure 6B with black arrowheads. The epithelial cells of the resin canals have thin cell walls, hence the earlywood area is visibly weak. Parenchyma ray cells close to the resin canals had a dark color. In these rays, chemical processes of the infection started to develop. A blue zone is visible in Figure 6C. The lumina in this zone are partially stained blue, probably because of the presence of nutrients that the bacteria need. Cavities in earlywood tracheid cell walls are visible in this figure, and are marked in the figure with white arrowheads. These cavities are the result of bacterial infection. Inside the lumina, growths are visible. Some growths were translucent (marked in the figure with black arrows), and they were not stained by any stain used. Some growths are dark, almost black (marked in the figure with white arrows), and these growths are also marked in Figure 6A. It is supposed that the translucent growths are the early stages of the bacterial infection process, and the dark growths are the end stages of the bacterial infection process. 

The dead spruce wood samples are presented in Figure 6D–F. In the dead spruce trees samples (Figure 6D), a blue stained zone was also found. The blue zones were located more towards earlywood than towards latewood. Earlywood tracheids have thinner cell walls than latewood tracheids. Earlywood tracheids also have more bordered pits than latewood tracheids. The main way in which the bacterial infection spread was through parenchyma ray cells and bordered pits, which is why this blue zone was located more in earlywood. In Figure 6E, dark oval-shaped growths are visible inside the lumina of the cell walls. These dark oval shapes on the cell walls in dead spruce tree samples did not have any growths inside the lumina. The growths inside the lumina were observed only in the healthy spruce tree samples. Figure 6F presents the radial cross-section of a dead spruce tree sample. A complete degradation of bordered pits in earlywood is visible. The tori are missing. The degraded bordered pits are now big pores in the earlywood cell walls. Missing tori disrupt physiological flow. They cannot fulfil their role of regulating the flow, which results in the infected tree losing moisture and drying out. Figure 6G presents how a healthy bordered pit should look.

**Figure 7:**
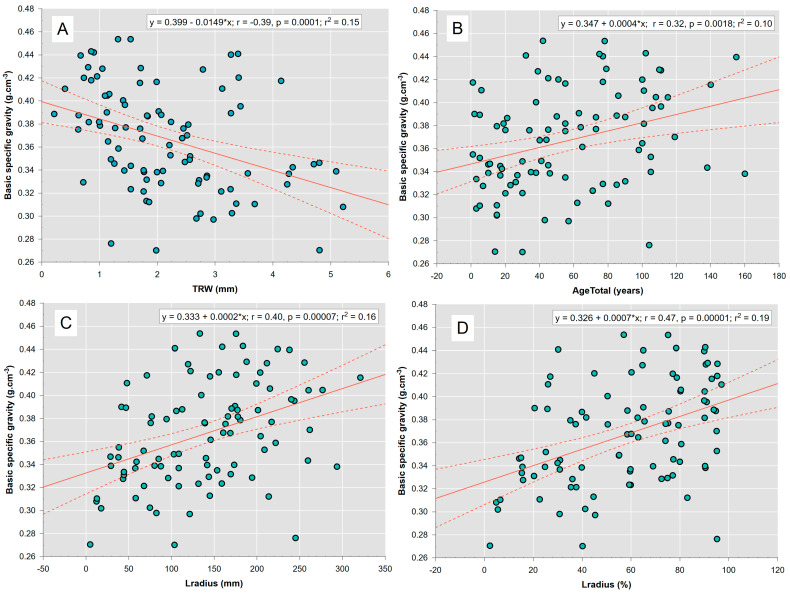
Regression analysis between growth characteristics and basic specific gravity of stems in the high mountain spruce forest. Point graphs were fitted with linear regression lines (red solid line) and corresponding regression bands (dashed red line).

There were no differences in the shape or strength of the correlations between groups of LTs and SDTs, so we pooled the values and presented them together in a single regression analysis for each growth characteristic (Figure 7). The values of stem basic specific gravity decreased with increasing tree-ring widths (Figure 7A). Higher radial increment values are associated with a decrease in the basic specific gravity of wood. The basic specific gravity increases with increasing age and tree size (stem diameter, Figure 7B–D). This is expected, since there is a biologically driven long-term growth trend of decreasing tree-ring increments associated with increasing tree age and size.

**Figure 8:**
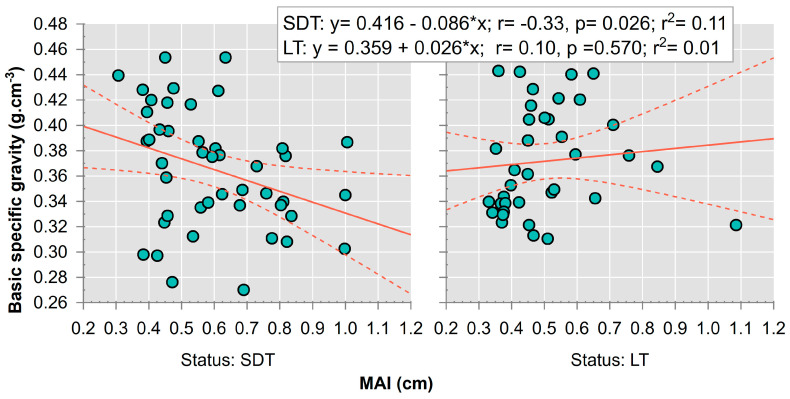
Regression analysis between mean annual increment (MAI) and basic specific gravity of stems in the high mountain spruce forest. SDT—standing dead tree, LT—living tree. Point graphs were fitted with linear regression lines (red solid line) and corresponding regression bands (dashed red line).

The mean annual increment, which is determined for each segment of the tree-ring series along the radial length, has a differential relationship with the values of the basic specific gravity of the SDTs compared to LTs (Figure 8). For the SDTs, a higher overall mean annual increment (higher mean growth rate) is associated with lower specific gravity values. Conversely, the mean annual increment (mean growth rate) of LTs has no relationship with specific gravity values.

**Figure A1:**
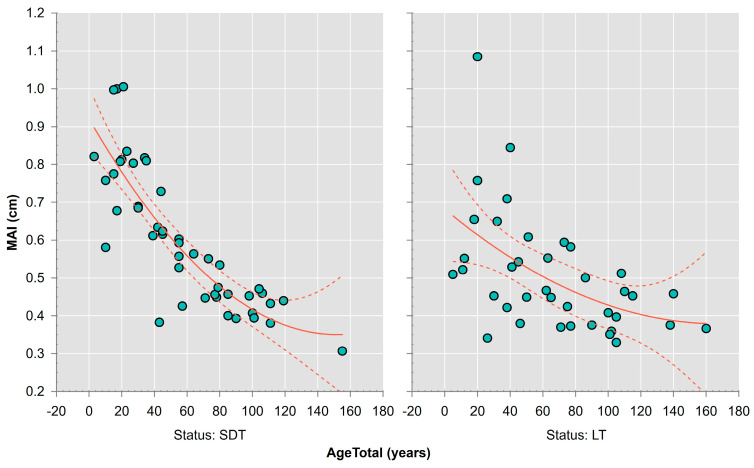
Mean annual increment (MAI) in relation to age (AgeTotal) according to groups of standing dead trees (SDTs) and living trees (LTs). The middles of split core segments were assigned with the corresponding variable value, retrospectively calculated from the TRW series based on the estimated AgeTotal and DBH values measured in the field. Point graphs were fitted with quadratic polynomial regression lines (red solid line) and corresponding regression bands (dashed red line).

**Figure A2:**
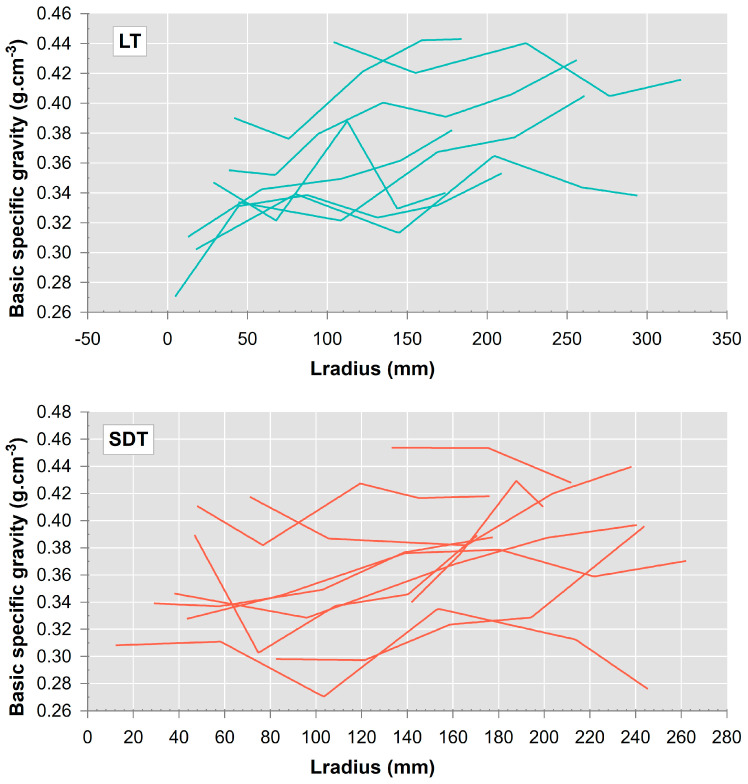
Radial variation of basic specific gravity of sampled living (LT) and standing dead (SDT) trees. “Lowess” smoothing was performed to fit the data.

The relationship between age and individual size (age–size relationships) is significantly less pronounced in the LTs compared to the SDTs. For instance, the age-related growth trend was not extracted, but TRW chronologies displayed a rather non-descending long-term trend (Figure 3). This is evident from the lower mean growth rate (MAI) at young ages and higher mean growth rate at later life stages of the LTs, as determined in retrospect for segments from the TRW series (Figure A1). As mentioned earlier, there was an increase in basic specific gravity values with an increase in diameter or radius, both in absolute and relative terms (Figure 7C,D). The validity of this trend was substantiated for the majority of individuals through the plotting of basic specific gravity values for individual trees along the radius (Figure A2). Initially, trees exhibited varying patterns of either decreasing or increasing basic specific gravity values near the pith, but in the final segments near the bark, the majority of trees displayed an increase in basic specific gravity values. Five trees were exceptions, three of which were in the SDT category. The decrease in basic specific gravity at the end of the radius in these individuals could not be accounted for by increased increments alone.

## 3. Discussion

High mountain spruce forests fulfil several important life-supporting ecosystem services [46]. During the baseline climate period (prior to 1990, as stated by the IPCC [47]), these forests exhibited stability and resilience in terms of their canopy tree crowns, which were protected from significant air pollution and strong winds. This stability was evident from low needle loss, high levels of resilience [48], signs of sustained growth, low mortality rates, and a gradual onset of natural regeneration [49]. Spruce forests exhibit a high level of adaptation to the environmental conditions found in high mountain regions, such as steep slopes with shallow soils, low temperatures, high air humidity, and a harsh and cold climate. 

Recently, fundamental components and the functionality of natural spruce ecosystems are facing a significant risk. Previous and recent observations have demonstrated their recent alterations in growth, especially outside the range of spruce natural distribution [50,51], and deterioration in health status [51], leading to increased mortality rates [52], although not consistently. The decline phenomena include dieback, decreased growth rates, mortality, and limited regeneration [53]. The general assumption is that the low secondary growth of declining trees is linked to carbon starvation and/or a failure in their hydraulic system [54] mechanisms, which underlie the predisposition of Norway spruce trees to subsequent biotic attacks [55]. 

In our study, the majority of sampled SDTs on the PO site showed symptoms of bark beetle infestation. Though bark beetles typically have low population levels and are limited in their ability to only attack trees that are weakened or dying [56], our results illustrate the mechanisms by which bacteria possibly associated with bark beetles induce a strong effect when attacking living trees with vigorous defenses [55,56]. The infestation process may begin in vital, dominant spruce trees, due to progressed bacterial infection (Figure 6A–C). 

We observed the following early signs of wood degradation: infection entering the lumina of the earlywood tracheids through bordered pits, the presence of cavities in earlywood tracheid cell walls, growths visible inside the lumina, the dark oval shapes on the cell walls (visible inside lumina in samples of neighboring dead spruce trees), and the complete degradation of bordered pits in earlywood, with missing tori causing infected trees to lose moisture and dry out (Figure 6D–F). The presence of bacteria in healthy trees can lead to a decrease in the functional area of sapwood and a change in the distribution of non-structural carbohydrates, thereby diminishing the trees’ capacity to withstand drought [7] and predisposing it to bark beetle attack.

The presence of favorable climate conditions has resulted in the enhanced growth of spruce trees [57,58]. However, the rise in temperature is also linked to the shifts of ungulates within different elevation ranges [59] and the increased likelihood of insect infestation, particularly bark beetles, following abiotic damage [60]. These factors have recently led to an unprecedented decline in the population of even young Norway spruce stands in the timberline zone. Wind scenarios in Central Europe indicate an increase in both frequency and speed [61], which, in turn, leads to an increase in the variety of disturbance types (agents). This raises doubts about the functionality of high mountain spruce forests, particularly in terms of enhanced carbon sequestration and storage [62]. 

In high mountains, the main disturbance factor is expected to be mechanical damage from snow or ice—prevailing over windthrown trees due to strong winds. In our study, SDTs accounted for 81% and 36% of the mortality at the PR and PO sites, respectively, representing a substantial part of the dead mature trees. More interestingly, the trade-off between the absolute number of dead trees and the proportion of standing and overthrown stems in the upper canopy layers was revealed. At the PO site, the higher overall number of dead trees in the canopy was reduced by a lower proportion of SDTs, and vice versa, the lower overall number of canopy trees was accompanied by a higher proportion of SDTs in the set of dead canopy trees at the PR site. Such a trade-off resulted in a very similar number (12–13 trees per hectare and decade) of SDTs in the canopy in both locations, which can serve as a very important “universal” input for the calculation of the intensity of intentional harvest interventions mimicking the natural dynamics of the stand. 

In high mountain spruce forests with moderate disturbance regimes, trees die continuously within a 10-year interval (9–12 years). The tending intensity per decade is 12–13 canopy trees per hectare, with a mean diameter reaching 50–58 cm (DBH > 33 cm). This corresponds to 2.5–4.5% of the total number of trees per hectare (DBH > 8 cm). The recommended share (55–60%) of trees with a diameter at breast height (DBH) greater than approximately 36 cm in the growing stock is required, to maintain the optimal (theoretical reverse J-shaped) diameter distributions in selection forests [63], as naturally spruce dominated forests exhibit various diameter structures of living trees [64].

Subsequently, based on the known proportion of standing and overthrown gap makers in the locality (stand, region), we can estimate the final tending intensity. For example, if we know that the percentage of standing gap makers out of all gap makers is 40%, we divide the minimum number of canopy trees (12–13) by 0.4, and we will receive a final tending intensity of 30–33 trees per hectare for a 10-year period, mimicking the true natural rate of gap formation by both fallen and standing dead trees (like the PO location).

Moreover, our study showed that the top diameter trees, which measure 70–80 cm, are recorded as logs, suggesting that they were uprooted (Figure 1). In even-aged forests, the typical pattern of tree rings is characterized by a decrease in width from the pith to the outer part of the stem, which is accompanied by a gradual increase in wood density [41]. Trees in uneven-aged forests exhibit a greater degree of variation in their ring-width patterns, based on the duration and intensity of tree suppressions and releases, thus displaying variable wood properties. In addition, long-developing trees in uneven-aged forests often have habitat features valuable for biodiversity, and the retention of old and dead trees with a special focus on biodiversity is recommended [65]. According to the two main certification schemes, FSC and PEFC, the retention of five habitat trees per hectare to live, which are mainly trees with large diameters, is prescribed. This is consistent with our results, where we found that dead top diameter trees are represented by numbers 1–4 stems per hectare.

Established after large-scale disturbance events and left without interventions, spruce forests of timberline zones (1200–1400 m asl) tend to form simple, single-layered, monospecific, and highly vulnerable structures [9,22]. Soil protection is often emphasized as the important function of these forests, with the aim to provide continuous shelter for forest ground to prevent erosion. The measures of vertical canopy projection potentially indicate the effectiveness of soil protection, because they relate to the capacity of forest canopy to intercept rainfall, reduce water drainage by litter and understorey, and bound soil particle movement by roots [66]. The total canopy cover (overlapping canopy cover) and utilization of potentially available space (growing space efficiency) reach the maximum values in the later phase of the grown-up developmental stage [67,68]. 

The maximum soil protective effect (prevention of soil erosion) of spruce natural forests could therefore be expected in the later phase of the grown-up developmental stage. The objective of protective management should then be to accelerate regeneration and recover the protection maturity [69]. Given the high cost and labor involved in maintaining and enhancing the protective effect, the aim of interventions should be to achieve a satisfactory level of soil protection rather than an ideal one, to guarantee the necessary functions of the protective forest for a period of 20 to 50 years. These interventions are collectively known as minimal tending of protective forest [70]. To mimic the small-scale dynamics and be successful, the initial phase of gap formation is the most important [71]. 

Based on our results, the presence and visual assessment of the first decay class of SDTs can serve to estimate the gap age (important for spatiotemporal models for minimum tending interventions) because the initial phase of gap formation has a narrow range of residence values. That means that if the SDTs in the first decay class are present in the gap, we have a 90–95% chance that the gap age is less than 10 or 13 years. At the same time, the mean residence time of 3 years, with a range of values between 2.6 and 3.6 years, indicates how often the regeneration processes initialized by the mortality of dominant and co-dominant canopy trees take place. In other words, if we count the number of gaps in a stand with a presence of SDTs, half of them were formed in the last 3 calendar years. This number divided by three determines the number of gaps formed each year, i.e., the rate of gap formation by natural mortality of standing canopy trees. Subsequently, based on the known proportion of standing and fallen gap makers, we can also estimate the rate of formations for all gaps (with or without the presence of SDTs). 

Larger and older trees have a tendency to reside (to persist) longer in the initial stage of gap formation. Standing dead trees typically undergo decomposition at a slower rate compared to wood in direct contact with the forest floor [25,34,72]. The presence of larger tree sizes or coniferous tree species resulted in SDTs persisting longer across large scales [35]. The diameter effect was not statistically confirmed in our study, due to the limited range of DBH values for adult canopy trees, similarly to results on decay in four North American tree species [44]. However, significant negative relations between the length of the radius, the age of the tree, and the tree-ring width with specific gravity of the wood support the idea of better persistence in larger and older trees. In fact, we can expect that trees with high specific gravity better withstand the pressures of mechanically acting abiotic factors. Due to this, there is a reasonable expectation that the enlargement of the sample size in natural conditions and the widening of the variation ranges of DBH and age will lead to statistical proof of their significant relation with residence time too.

Slow growth rates in high mountains are associated with higher density and top-quality timber, and therefore with the higher economic value of old trees. Unlike in even-aged forests (e.g., naturally established after large-scale disturbance), uneven forest structure and management can improve carbon uptake, and thus also the mitigation function, because it ensures that the forest structure remains constant, and therefore allows for sustainability [73].

The values of wood specific gravity to calculate the biomass of standing trees (around 0.450 g.cm^−3^ at a wood moisture content between 12 and 15%) are different from the data we found, which suggests that the calculations tend to overestimate the biomass. Similarly, with our data, other research from various areas of Slovakia and surrounding countries reports values around 0.370–0.380 g.cm^−3^ [74,75], but not exclusively [76,77]. In addition, both the intra-tree and inter-tree variability of the observed values of basic specific gravity is high and, in our study, ranged from 0.302 to 0.523.

Although on average basic wood specific gravity did not differ between groups of LTs and SDTs (Table 2), it can be concluded that in both groups the amount of cell wall substance may decrease on the outside part of the stem (near the bark) and thus, wood decomposition due to microorganisms may occur (Figure A2). Therefore, the retention of SDTs in their current state leads to the storage of carbon that is equivalent to that of living trees for a duration of 9–12 years, with an average of 3 years. The relationships between tree-ring width and wood properties are well known. The species-specific width of the latewood in conifers remains relatively constant, despite the observed variability in the total ring width [42]. Thus, larger tree rings with a smaller proportion of latewood display a smaller density [78].

The ring width, age, and size of trees were significantly related to basic wood specific gravity (Figure 7). However, the mean annual diameter increment was the discriminant factor between SDTs and LTs (Figure 8). The presented findings can probably be explained by the different shapes of the increment curves between the SDTs and LTs. The SDT group is dominated by individuals that show high increments during the early-growth phase, and a peak followed by a decline at a relatively young age (approx. 35 years). We assume that most of these individuals were established as open-grown trees (Figure A1). LTs were more commonly established under the canopy. Individuals have growth associated with rather low MAI values at the onset of growth and at small dimensions.

## 4. Materials and Methods

### 4.1. Study Area

In the high mountain ranges of Slovakia, a continuous belt of spruce-dominated forests represents the upper timberline (approx. 1200–1400 m asl) and tree line (>approx. 1400 m asl) zones. Spruce dominance is characterized by an ability to withstand harsh and cold mountain climates in varying geomorphological conditions. Yet, on some high mountain ridges, the upper tree line is represented by a beech belt, and in others, beech enters stands under spruce canopy (up to approx. 1200–1300 m asl, rarely > 1400 m asl). Other admixed tree species are rowan, sycamore and birch. The overall characteristics of high mountain ranges are 2–4 °C mean annual temperature, 1100–1600 mm annual precipitation sum, short growing seasons lasting 2–3 summer months, and long durations of snow cover up to 5–6 months [79].

**Figure 9:**
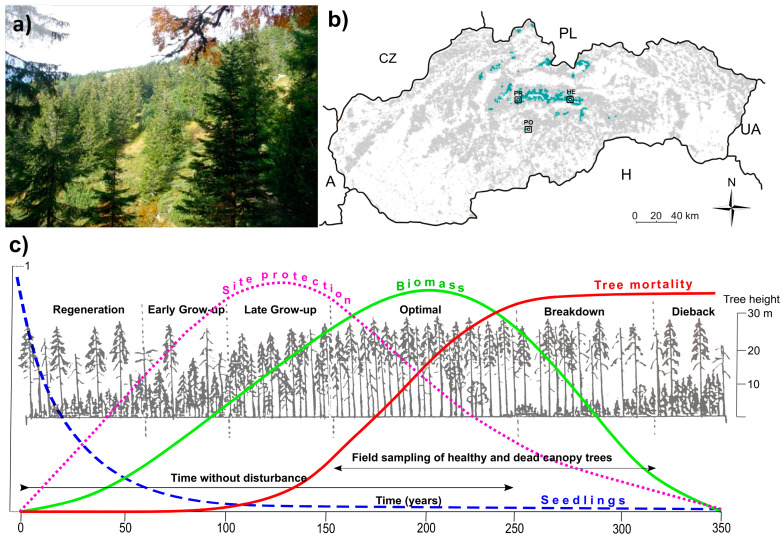
(**a**) Study site Prašivá (PR) on a steeper slope. (**b**) Location of sampled study sites in Slovakia, PO (Poľana Mt.), HE, PR (Heľpa, Prašivá, Low Tatra Mts.). Squared are localities with repeated inventory. High mountain spruce forests (green color) constitute approx. 1.5% of total forest cover (grey). (**c**) Schematic development of soil erosion protective effect, biomass, number of seedlings, and probability of tree mortality of individual canopy trees during five developmental phases of a single tree cohort in Norway spruce forest at high mountain timberline zone as adopted from [8,9]. Note that in the scheme, the breakdown and dieback phase of one cohort is mixed with regeneration and early grow-up phase of another cohort; the core sampling of dominant and co-dominant canopy trees for this study was conducted in optimal and breakdown developmental phases.

In these natural conditions, a network of permanent research plots has been established within the framework of a long-term study of natural forests’ dynamics, where, among others, the occurrence and structure of dead wood in the sense of coarse woody debris (CWD, diameter > 8 cm) has been recorded for more than 30 years. In addition, our department has recently studied spruce growth under natural conditions to investigate the processes of natural (background) mortality of individual spruce trees or small groups of trees. At present, there are relatively few sites where the growth of high mountain spruce trees has been undisturbed for a substantial length of time, i.e., without the impact of large-scale wind and subsequent bark beetle disturbances, or without being exposed to heavy air pollution in the past. Three localities that fulfil the above criteria were selected for the purpose of this study: Helpa (HE) and Prasiva (PR) in the Low Tatra Mts., and Polana (PO) in Polana Mt (Figure 9).

### 4.2. Deadwood Analysis

In the two localities of PO and PR, the mortality of trees was analyzed from repeated inventories. The intensity (number of dead trees per hectare) of mortality between two consecutive inventories was recalculated and expressed for the 10-year period. In the PR protection forest, 31 circular permanent research plots (PRP, 400 m^2^) were established in a regular grid (56.5 m) in 2007, and were subsequently remeasured in 2022. In the PO old-growth forest, 20 circular PRPs (1000 m^2^) were placed in a regular grid (100 m) in 2013, and remeasured in 2018. The intensity (number of dead trees per hectare) of mortality between two consecutive inventories was recalculated and expressed for the 10-year period. The minimum DBH of standing dead trees (33 cm in both localities) with dominant and co-dominant social positions was set to separate canopy mortality from mortality of trees resulting from auto-reduction processes under the canopy. The locality of HE was not included in deadwood analysis, because the measurements of LT and SDT characteristics did not come from repeated inventories.

On each plot, the diameter at breast height (DBH) of all living and standing dead trees and snags was measured (diameter > 8 cm). Results on deadwood structures were analyzed from the last (2018 and 2022) inventory. The diameter at both ends (thicker—d_0_ and thinner—d_n_) and stem length (L) of the lying deadwood were measured. Tree and snag heights were also recorded. Tree heights were employed for modeling the height, using the Prodan and Michajlov height curves. Two parameter (DBH, height) equations by Petráš and Pajtík [80] were used to compute the volume of living (V) and standing dead trees and snags (V_s_). Stand density (N—number of living trees per hectare) and respective stand basal area (BA) were calculated for all living trees. To calculate the snag volume, we assumed the direct proportionality of trunk thickness at different heights. The lying deadwood volume (V_l_) was calculated using Smalian’s formula:

(1) $$V_{l}=\frac{\pi }{4}\times \frac{\left(d_{0}^{2}+d_{n}^{2}\right)}{2}\times L$$

Total deadwood volume (V_d_) expressed in m^3^.ha^−1^ is sum of V_l_ and V_s_.

### 4.3. Analysis of Standing Dead Trees

Dominant abiotic factors vary along a gradient of environmental conditions. At high altitudes, abiotic factors such as snow or frost have a major influence on mortality. Trees die standing primarily due to mechanical damage (frequent occurrence of multiple stem and crown breaks), followed by the impact of mostly biotic pests. The decay classes used in the long-term inventory of natural (old-growth) forests in Slovakia follow a four-class system, with decay class one being least decayed [81]. Standing dead trees (SDTs) in the first decay class are assigned based on the visual inspection of the presence of tree morphological features, namely the presence of needles, fine twigs, and branches, intact sapwood with minimal decay, the presence of the top of the crown, and all bark remains. Gap makers in the initial phase included in our analysis are SDTs with dominant or co-dominant social positions within the stand. Downed dead trees, or stumps from above-ground coarse woody debris, were not sampled. A tree was considered dead if it had 100% needle loss. 

### 4.4. Dendrochronological Dating of LT and SDT Core Samples

In the three localities PO, PR, and HE, we sampled Norway spruce trees for growth analysis during 2019–2023. At these sites, we randomly selected plots approximately every 200–300 m, and identified a small gap formed by a dead canopy tree—a standing dead spruce with decay class one (SDT) and a vital (defoliation < 35%) spruce with a dominant position (the largest diameter of the 10 nearest trees). 12–18 plots were established at each site.

For dating purposes, 12–18 living dominant trees (LTs) were sampled to build mean (master) site chronologies. From each sampled SDT or LT, one increment core was extracted at breast height (1.3 m) at a 45° angle to the slope direction, to avoid sampling reaction wood. In the PO locality, two cores per stem were taken from SDTs and LTs perpendicular to the slope direction from one side, and from the opposite side of the stem for the subsequent determination of wood basic specific gravity. DBH was measured by the calliper following the increment coring direction. Increment cores were sealed in the plastic rows and transported to the laboratory. For cross-dating and tree-ring measurements, the cores were placed into 5 mm wooden bars, air dried, mounted, and sanded with progressively finer sanding paper, until wood cells became clearly visible under the microscope.

Tree-ring widths (TRW) were graphically cross-dated and measured to an accuracy of 0.01 mm, using the software CooRecorder (Cybis Elektronik & Data AB, Saltsjöbaden, Sweden, [82]). The cross-dating of tree-ring series for the establishment of master (mean) chronologies was checked in TSAP software (Rinntech, [83]), and by using the software COFECHA [84]. Mean chronologies were developed by averaging the TRW measurements from each calendar year with bi-weight robust mean. Chronologies were truncated to sample depth > 5 and drawn for each locality. Similarly, the basic dendrochronological characteristics of tree-ring series were calculated for each locality. All calculations were performed in the R-environment [85] using the package dplR [86].

The calendar year of SDT death—the last formed tree-ring or partial ring—was successfully set by cross-dating the measured TRW series of SDTs with established mean chronologies. For each TRW series of LTs and SDTs with the pith not presented on the core, the missing years to pith (YTP) at the coring height of 1.3 m were determined by graphical estimation, by fitting the concentric circles to the curves of the inner arcs [87]. Distance to pith (DTP), e.g., missing radius, was also recorded. The whole core (tree) pith age (AgeTotal), DBH, and mean annual increment (MAI) according to groups of trees were summarized and tested for differences (Table 3).

### 4.5. Estimation of the Mean Time of Gap Creation (Formation)

Based on the dendrochronological dating of single TRW series, we obtained the dataset of SDTs assigned by years since their death. The value of one year was assigned to recently dead trees in first decay class, if trees died in the growing season (partially formed last ring or the complete tree-ring measured in distance from bark to pith). The Kruskal–Wallis ANOVA and Median test was used to assess the differences among sampling localities in mean time since death, measured in years. The results of the test confirmed that the samples considered were drawn from the same distribution (with the same median) and were therefore grouped into one sample.

The frequency table of times since death was produced on a merged dataset, with 12 intervals with a length of 2 years, and cumulative relative frequencies were calculated. The complement of the cumulative distribution (1 − x) was fitted by the exponential function, to model the distribution of residence times of SDTs in the first decay class:

(2) $$t(1/2)=1e^{-k\;yrs}$$

The parameter k (*p* < 0.01) of the function was estimated from empirical data, using the nonlinear least-squares criterion and the Levenberg–Marquardt algorithm, and was used to reliably determine the half time of the residence of SDTs in the first decay class (mean time of gap formation; Equation (3)):

(3) t(1/2)=ln⁡(0.50)/k

Complementary cumulative distribution is interpreted as the distribution of ‘survival’ (residence) time of SDTs in the first decay class. For example, the functional value of 1 year, for the first interval (0–2 years), means that 100% of the times since death in a considered SDT set were higher than the lower border of the first interval 0, which means that all SDT times since death were longer than 0. Subsequently, the functional value 0.5 for the second interval (2–4 years) means that 50% of SDT times ‘survive’, i.e., they are more than 2 years old, and 50% of SDTs stay in the first decay class for longer than 2 years, etc.

### 4.6. Basic Specific Gravity

The specific gravity of wood in trees can be estimated from samples taken with increment borers [88]. The technique is carried out by extracting increment cores from trees and measuring their volume and mass in a laboratory [88,89]. In this study, basic specific gravity (ρ_B_) is determined for the SDTs and LTs in the PO locality, where two cores per tree were collected. Each second core protected from moisture loss by careful wrapping into plastic straws was processed in the laboratory, after a short cold storage at 4 °C. Cores were carefully split into several segments (n) of similar lengths, yielding in total 100 samples, thus also allowing for the examination of radial (internal) ρ_B_ distribution [90]. All core samples taken from the stems had either pith presented on the core, or the distance to pith (DTP—missing part to pith) represented, on average, less than 4% of the radius length and, on average, less than two missing years to pith (YTP). We assumed that missing DTP or YTP had a negligible effect on the calculation of the whole core ρ_B_ value (Equation (4)), reported in this study as an estimate of tree ρ_B_ at 1.3 m height.

(4) $$\rho_{B}=\frac{\sum_{i=1}^{n}\rho_{Bn}}{n}$$

The initial and oven-dried dimensions of the samples were measured to 0.01 mm and weighed to 0.001 g. The volume of each sample (Equation (5)) was determined as the length of the sample multiplied by the surface of the base:

(5) $$V_{M(0)}=l\;3.14\;{\left(\frac{5}{2}\right)}^{2}$$

V_M_ and V_0_ = sample initial and oven-dried volume expressed in mm^3^, l = length of the sample in mm.

Samples were dried in an oven with good air ventilation at 103 °C ± 2 °C for 24 h to a constant weight. The basic specific gravity, ρ_B_ (Equation (6)) was determined as samples’ oven-dried mass divided by its initial volume.

(6) $$\rho_{B}=\frac{m_{0}}{V_{M}}$$

To determine the possible mass loss of SDTs in the first decay class, we tested the differences in the whole core ρ_B_ values between LTs and SDTs. The *t*-test was used to evaluate the differences in means between groups of SDTs and LTs.

### 4.7. Wood Anatomy

To identify early signs of wood decomposition, two SDTs were selected with the highest (ρ_B_ = 0.523, sample S01) and the lowest (ρ_B_ = 0.302, sample S04) basic specific gravity values, and from the same sampling plot corresponding to LTs (ρ_B_ = 0.334, sample H01; ρ_B_ = 0.388, sample H04). 

Two small samples were cut from the selected extracted cylindrical wood fragments (the last segments of the tree core). The small samples were embedded in epoxy resin and left to cure for two weeks. Approximately 15 μm-thick microsections were cut from the wood–resin blocks on a sledge microtome (Reichert, Vienna, Austria). The microsections were stained with a combination of Astra Blue and Safranin stains. The Astra Blue and Safranin stain combination can also be used to distinguish cellulose from lignified cells [91]. Safranin stains lignin regardless of whether cellulose is present, whereas Astra Blue stains cellulose only in the absence of lignin [92,93]. A combination of Safranin and Astra Blue is used to detect early stages of white rot, i.e., selective delignification [92]. After staining, the microsections were mounted in Euparal (BioQuip Products Inc., Rancho Dominguez, CA, USA).

### 4.8. Basic Specific Gravity and Radial Variation

For possible differences in SDTs and LTs in age, size, and growth-related characteristics, we analyzed the differences in the variation of basic specific gravity values determined along the radius, and the relationships to selected variables for each core segment, separated by groups of SDTs and LTs. The variables DBH, TRW, MAI, absolute (Lradius) and relative length of the radius (Lradius(%)), and AgeTotal entered regression analysis as independent variables. The middle of each segment was assigned the corresponding ρB value, and the values of the above-selected variables. The variables corresponding to the segment were retrospectively calculated from the TRW series, based on the AgeTotal and DBH values measured in the field.

The relationships were examined by linear regression analysis, after testing data for their normal distribution and removing the outliers from the dataset. To supplement the findings, the graphical display of the AgeTotal and MAI values is provided in Figure A1. To complete the information, graphs illustrating the radial variations in ρB per tree and the corresponding trends obtained by “lowess” smoothing are also supplemented (Appendix A).

## 5. Conclusions

The Norway spruce appears to possess a unique capacity for slow-developing natural regeneration beneath the canopy of standing trees or in small gaps. Considering the correspondence of the single standing dead trees in the first decay class with the initial phase of gap formation, our study quantified the amount, intensity, and time interval of possible minimum interventions for management guidelines, which are scarce and rarely available data in mountain stands undisturbed by large-scale disturbances. More specifically, the approach for estimating the rate of gap formation and the number of canopy trees per area unit needed for their intentional formation, was formulated based on residence time analysis and observations of a rather invariant absolute number of spruce trees dying standing in canopy trees in different localities. Thus, new findings on the initial phase of gap formation provide a basis for the objective proposal of intervals and intensities of tending interventions, designed to mimic the small-scale dynamics of natural stands.

In addition, interesting facts about the cause and impacts of tree death on deadwood density in the initial phase gap formation were revealed. Especially, the absence of fungal and prevalence of bacterial infestations in the wood, the presence of bacterial infestation already in living trees, and the lack of difference among the specific gravity between standing dead and living trees were surprising. That suggests that one of the possible explanations for the slower rate of wood decomposition in mountain conditions is bacterial infestation, and that the process of wood decomposition is the long-term process, starting even in living trees. At the same time, it seems that standing dead trees may be harvested for use in products with longer-term carbon storage. In such cases, they must be processed no later than up to 3–10 years from death.

The stand structure in high mountain spruce forests at the subalpine belt, with the extreme climatic conditions of Central-East Europe, is characterized by an uneven structure, a feature that is also observed in natural primeval forests. In the timberline zones of Norway spruce-dominated forests, interventions may be necessary to improve the health and ecological stability of the mountain spruce stands, and to sustain the uneven-aged structure (either by selection or group selection cuttings). Additionally, the retention of old and dead trees, with a particular focus on biodiversity, is highly desirable. A proper intentional silviculture of mountain spruce stands will have increasing importance in the future, due to the pronounced negative effects of climate change and the urgent need to secure soil protection and carbon sequestration in extreme mountain conditions.

## Figures and Tables

**Table 1:** Basic characteristics of Norway spruce-dominated stands and deadwood.

Site	Year	Living Trees			Deadwood		
		N	BA	V	V_l_	V_s_	V_d_
		Stems·ha^−1^	m^2^·ha^−1^	m^3^·ha^−1^	m^3^·ha^−1^	m^3^·ha^−1^	m^3^·ha^−1^
PO	2018	254 ± 21	39.1 ± 2.5	375 ± 27	143 ± 17	22 ± 6	165 ± 20
PR	2022	464 ± 41	35.0 ± 2.5	315 ± 29	56 ± 9	57 ± 11	113 ± 16

Note: Numbers are expressed as means ± standard deviations. Spruces accounted for 96% of the number of living trees (N, DBH > 8 cm) in PO (Polana) and PR (Prasiva). Year—the year of last census of trees, BA—basal area, V—volume, V_l_, V_s_, V_d_—lying, standing and total deadwood volume (diameter > 8 cm).

**Table 2:** Basic specific gravity and corresponding length of the stem radius in the high mountain spruce forest.

Variable	LT	SDT	t	*p*
	Mean ± SD	Mean ± SD		
LRadius (mm)	251.25 ± 53.21	235.54 ± 42.36	–0.820	0.421
Mean basic specific gravity ρ_B_ (g.cm^−3^)	0.369 ± 0.041	0.380 ± 0.061	0.413	0.685

Note: Numbers correspond with means and standard deviations (SD) for 13 standing dead trees (SDTs) and 12 living trees (LTs), LRadius—length of the stem radius.

**Table 3:** Growth characteristics of living and dead Norway spruce trees.

Site	Variable	LT	SDT	t	*p*
		Mean ± SD	Mean ± SD		
PO	AgeTotal (years)	127 ± 18	124 ± 20	–0.328	0.746
	DBH (cm)	50.3 ± 10.6	47.1 ± 8.5	–0.820	0.421
	MAI (cm)	0.395 ± 0.056	0.382 ± 0.064	–0.542	0.593
	**nT/C**	12/24	14/28		
PR	AgeTotal (years)	145 ± 36	175 ± 62	–1.659	0.108
	DBH (cm)	57.4 ± 13.2	48.6 ± 14.8	1.641	0.112
	MAI (cm)	0.408 ± 0.107	0.313 ± 0.192	1.395	0.174
	**nT**	18	12		
HE	AgeTotal (years)	97 ± 31	123 ± 23	–1.602	0.127
	DBH (cm)	45.2 ± 6.8	41.7 ± 16.2	0.634	0.534
	MAI (cm)	0.516 ± 0.186	0.328 ± 0.067	2.099	0.050
	**nT**	15	9		

Note: PO—Polana, PR—Prasiva, HE—Helpa, LT—live tree, SDT—standing dead tree, SD—standard deviation, t—test criterion, and *p*—significance values. AgeTotal, DBH, MAI—tree age, diameter, and mean annual increment at 1.3 m height, nT/C—number of sampled trees/cores.

## Data Availability

The raw data supporting the conclusions of this article will be made available by the authors on request. The data are not publicly available due ethical restrictions.
